# Admitted AIDS-associated Kaposi sarcoma patients: Indications for admission and predictors of mortality

**DOI:** 10.1097/MD.0000000000022415

**Published:** 2020-09-25

**Authors:** Faheema Vally, Wencilaus Margret Pious Selvaraj, Owen Ngalamika

**Affiliations:** Dermatology and Venereology Division, University Teaching Hospital, University of Zambia School of Medicine, Lusaka, Zambia.

**Keywords:** Admitted, HIV-associated, Kaposi sarcoma, mortality, predictors, sepsis

## Abstract

Kaposi sarcoma (KS) is an AIDS-defining angioproliferative malignancy associated with high morbidity and mortality. Most KS patients in regions with high incidence such as sub-Saharan Africa present late with advanced stage disease. Admitted KS patients have high mortality rates. Factors associated with mortality of admitted KS patients are poorly defined.

We conducted a retrospective file review to ascertain reasons for admission and identify factors associated with mortality of admitted HIV-associated (epidemic) KS patients in Zambia. Baseline study variables were collected, and patients were retrospectively followed from admission to time of discharge or death.

Mortality rate for admitted epidemic KS patients was high at 20%. The most common reasons for admission included advanced KS disease, severe anemia, respiratory tract infections, and sepsis. The majority (48%) of admitted patients had advanced clinical stage with visceral involvement on admission. Clinical predictors of mortality on univariate analysis included visceral KS [odds ratio (OR) = 13.74; 95% confidence interval (95% CI) = 1.68–113; *P* = 0.02), fever (OR = 26; 95% CI = 4.85–139; *P* = .001), and sepsis (OR = 35.56; 95% CI = 6.05–209; *P* = .001). Baseline hemoglobin levels (5.6 vs 8.2 g/dL; *P* = .001) and baseline platelet counts (63 x 10^9/L vs 205 x 10^9/L; *P* = .01) were significantly lower in mortalities vs discharges. Baseline white cell counts were higher in mortalities vs discharges (13.78 x 10^9/L vs 5.58 x 10^9/L; *P* = .01), and HIV-1 viral loads at the time of admission were higher in mortalities vs discharges (47,607 vs 40 copies/μL; *P* = .02). However, only sepsis (or signs and symptoms of sepsis) were independently associated with mortality after controlling for confounders.

In conclusion, common reasons for admission of epidemic KS patients include advanced disease, severe anemia, respiratory tract infections, and signs and symptoms of sepsis. Signs and symptoms of sepsis are independent predictors of mortality in these patients.

## 1 Introduction

Kaposi sarcoma (KS) is an angioproliferative spindle cell tumor of endothelial origin.^[1]^ It is caused by the Kaposi sarcoma herpesvirus (KSHV).^[2]^ KSHV seroprevalence is high in sub-Saharan Africa, and hence the high prevalence of KS in this region.^[3]^ KS commonly not only affects the skin, but can also affect other parts of the body, including the gastrointestinal tract, respiratory tract, lymph nodes, and conjunctiva of the eyes.^[1]^

There are 4 main subtypes of KS. These include classic, endemic, iatrogenic, and epidemic KS.^[4]^ Classic KS mainly affects elderly men of Eastern European descent. Endemic KS, also known as African cutaneous KS, is more common in sub-Saharan Africa among HIV-seronegative individuals. Iatrogenic KS mainly affects patients on immunosuppressive drugs such as those undergoing organ transplants. Epidemic or AIDS-Associated KS is seen in HIV-infected individuals, is a WHO HIV stage 4 disease, is the most common type of KS worldwide, and has a rapidly progressive disease course.^[4]^

The introduction of antiretroviral therapy (ART) has led to a reduced incidence and improved survival of epidemic KS globally.^[5]^ However, despite combination antiretroviral therapy, epidemic KS is still highly prevalent with high mortality rates world-wide, and especially in sub-Saharan Africa.^[6]^ Between 2008 and 2013, the University Teaching Hospital (UTH) in Zambia recorded at least 726 incident KS cases, majority of which were HIV-associated.^[7]^

Unlike other forms of KS, AIDS-associated KS has a rapidly progressing disease course, and may disseminate in a short period of time to involve the viscera, including lungs and gastrointestinal tract. ^[5]^ About 10% to 20% of patients present with advanced disseminated disease and/or complications such as anemia, and are admitted to the inpatient medical wards. Several factors including stage of KS at presentation, ART status, and CD4 counts have been reported to predict outcomes of KS patients.^[4]^ In addition, recurrence rates of chemotherapy-treated epidemic KS patients are high.^[8]^

KS remains one of the most common AIDS-related malignancies in Zambia, with numerous admissions to our hospitals. KS patients make up about 70% of all dermatological admissions at UTH. There is paucity of data regarding the factors affecting outcomes of admitted epidemic KS patients. Most previous studies have focused on outcomes of epidemic KS in general, and not specifically on the admitted population of epidemic KS patients. Therefore, this study aimed at determining reasons for admission, and identifying clinical and laboratory parameters that predict mortality or discharge of admitted epidemic KS patients.

## 2 Methods

A retrospective file review of a series of HIV-associated/epidemic KS patients admitted in the period from April 2019 to November 2019 was carried out. We reviewed medical records of adult epidemic KS patients aged 18 years and older, who were admitted to the medical wards of the Adult hospital of the University Teaching Hospitals (UTH) in Lusaka, Zambia.

We used printed data collection forms to obtain information from patient files. The information was later entered into an excel spreadsheet and then exported to STATA for analysis. The clinical and sociodemographic information collected included age, gender, previous hospital admissions, smoking, alcohol use, ART status, adherence to ART, duration on ART, other comorbidities (e.g., tuberculosis, sepsis, diabetes, hypertension, hepatitis B), duration of KS, KS clinical stage, history of receiving chemotherapy, fever, palor, jaundice, weight loss, treatment given during admission (chemotherapy, blood transfusion, oxygen, antibiotics, ART). We also collected laboratory parameters, including full blood count, liver function test, renal function test, HIV viral loads, CD4 counts, and histopathology staging.

The main objective of the study was to identify reasons for admission and factors associated with outcomes (discharge or mortality) of admitted adult epidemic KS patients at UTH. Ethical approvals for a waiver of consent were obtained from the University of Zambia Biomedical Research Ethics Committee, and the National Health Research Authority. Obtaining informed consent from the actual participants was not possible, as this was a retrospective study and study participants were not available to give consent, as they were either discharged or had died.

All data were analyzed using STATA version 15 (StataCorp, TX). Baseline characteristics were analyzed using summary and descriptive statistics. Continuous variables are presented as median and interquartile range, while dichotomous or categorical variables are presented as percentages. The Mann–Whitney test was used for group comparisons of continuous variables. Univariate logistic regression was used to determine the association between the dichotomous outcome and the categorical and continuous predictors. Multivariate logistic regression was used to control for confounders. Predictors with *P* values < .25 on univariate logistic regression and predictors known to be associated with the outcome regardless of *P* values, were included on the multivariate logistic regression model. Among the highly correlated variables, only one was included in the multivariable model. The prediction of our model for outcome was determined by the AUC-ROC curve. The Hosmer–Lameshow goodness of fit test was used to determine how well our model fit the data. We also used the logistic regression model to test for interactions between variables that may possibly modify the observed effect.

## 3 Results

We collected and analyzed information of 54 epidemic KS patients admitted in the period from April 2019 to April 2020. Among these, 43 (80%) were discharged, while 11 (20%) were mortalities. Baseline characteristics of the study participants, by outcome, are summarized in Table 1.

**Table 1:**
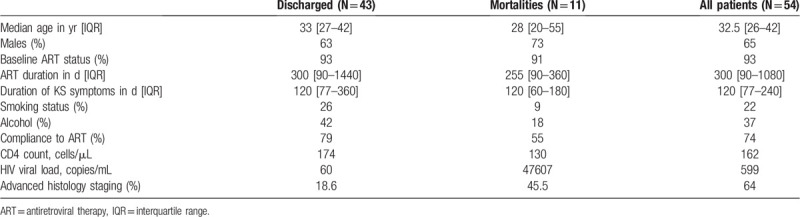
Baseline characteristics of admitted KS patients by outcome.

**Figure 1:**
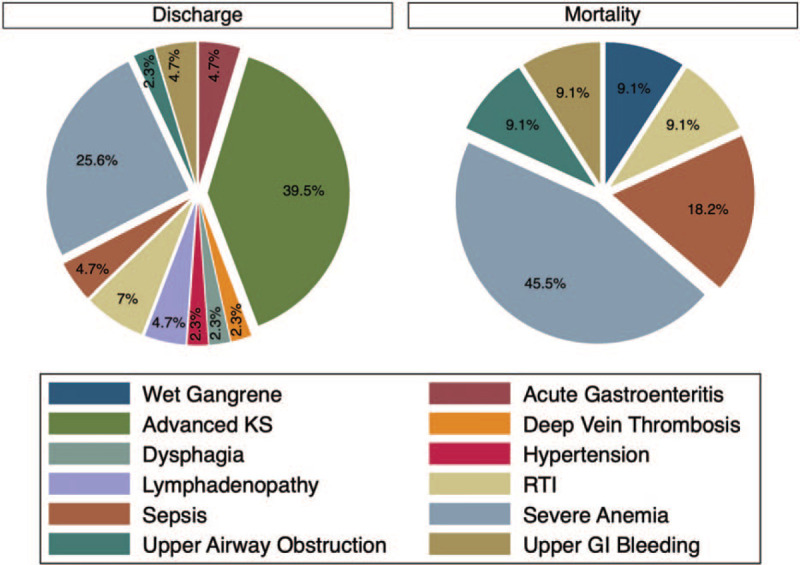
Admission diagnoses by outcome. Advanced KS and severe anemia were the most common admission diagnoses among the patients who were discharged. Severe anemia and sepsis were the most common admission diagnoses among the patients who died in the hospital. GI = gastrointestinal, RTI = respiratory tract infections.

The most common reason for admission for discharged patients was advanced/disseminated KS followed by severe anemia, and the most common reason for admission for individuals who died was severe anemia followed by sepsis (Fig. 1). Other reasons for admission in all the patients included respiratory tract infections, acute gastroenteritis, upper gastrointestinal bleeding, upper airway obstruction, dysphagia, hypertension, deep vein thrombosis, wet gangrene, and generalized lymphadenopathy.

KS clinical staging at presentation was different between the discharged patients and mortalities. Among the discharged patients, 14% presented with localized cutaneous KS, 49% had disseminated cutaneous KS, while 37% had advanced KS (cutaneous along with visceral KS). Among the mortalities, 9% had disseminated cutaneous disease on admission, while 91% had advanced KS.

On univariate logistic regression, the factors significantly associated with mortality included history of previous hospital admissions, presence of visceral KS, fever, and an admission diagnosis of sepsis. In addition, hospital interventions including oxygen therapy, intravenous antibiotics, and blood transfusion were significantly associated with mortality (Table 2).

**Table 2:**
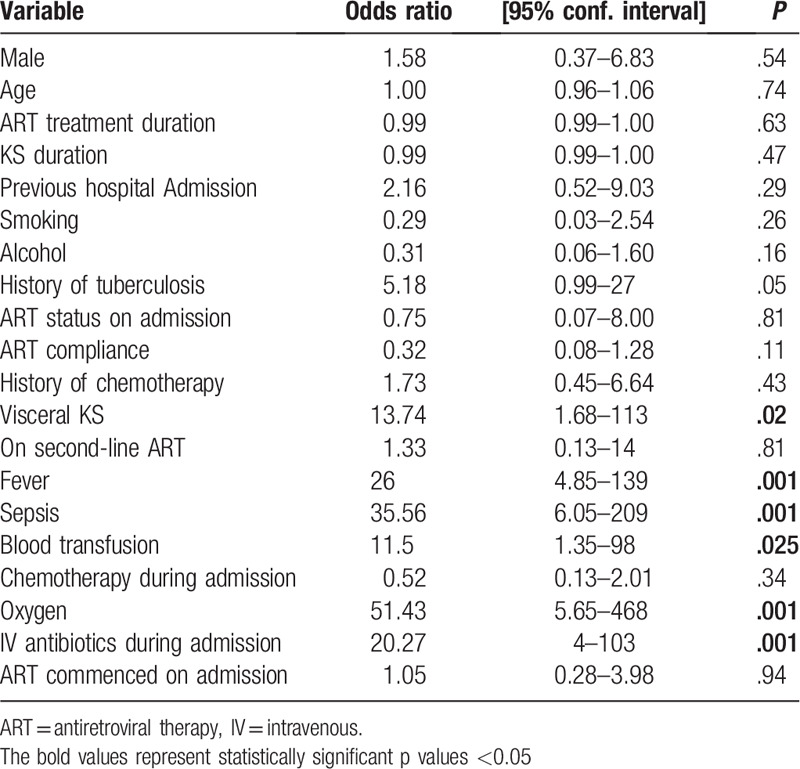
Univariate logistic regression analysis for clinical predictors of mortality.

We also collected and compared results of routine laboratory tests done on admission. Individuals who died had statistically significantly low hemoglobin levels and low platelet counts as compared to individuals who were discharged. Those who died also had statistically significantly high white blood cell counts and median HIV viral load as compared to those who were discharged (Table 3).

**Table 3:**
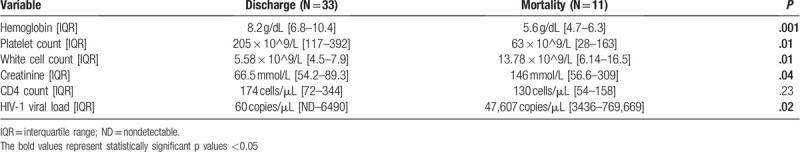
Association between laboratory parameters and outcomes.

We built a multivariate logistic regression model with variables that were statistically significant on univariate logistic regression and the laboratory parameters that were significantly associated with the outcome on univariate analysis. After adjusting for age, gender, hemoglobin levels, platelet count, and creatinine, sepsis remained a significant independent predictor of mortality (Table 4). The multivariable logistic regression model was a good predictor of mortality with an area under the curve of 0.98. The model was also a good fit according to the Hosmer–Lameshow goodness of fit test (*P* = .96). In addition, there was no multicollinearity of the variables in the model, with a mean variance inflation factor of 1.36 (highest = 1.57, lowest = 1.13).

**Table 4:**
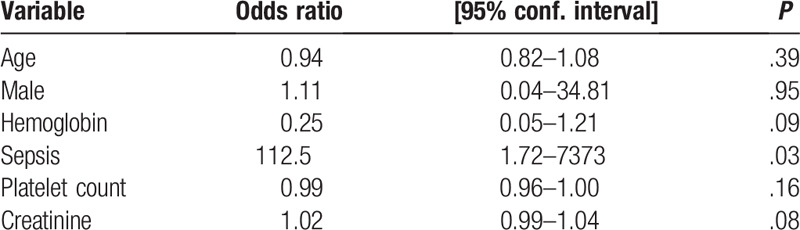
Multivariate logistic regression for predictors of mortality.

Testing for interactions between sepsis and intravenous antibiotics (*P* = .30), and between blood transfusion and anemia (*P* = .69) were not statistically significant.

## 4 Discussion

This study evaluated the factors associated with mortality of admitted adult epidemic KS patients at UTH, in Zambia. Twenty percent of all our admitted KS patients during the study period died in the hospital. This is similar to a prospective 10-year study conducted in our neighboring country, Tanzania, where a mortality of 24.2% was reported.^[9]^ However, the Tanzanian study included all KS patients regardless of hospital admission status.

Common reasons for admission among patients who were eventually discharged included disseminated/advanced KS and severe anemia, whereas the most common reasons for patients who eventually died were severe anemia and sepsis. Advanced HIV disease is in itself a risk factor for developing anemia and/or sepsis.^[10,11]^ In addition, KS has been previously reported to be associated with severe anemia.^[12]^ It is therefore not surprising that anemia and sepsis were among the most common reasons for admission.

Late KS presentation with advanced disease on admission was a common feature in both the discharged patients and in those who died. However, those who died had a higher proportion of individuals with advanced disease than those who were discharged. This is similar to a study conducted in another sub-Saharan African country where a majority (69%) of AIDS-associated KS patients presented with advanced disease and had high mortality rates.^[13]^ These findings suggest that presentation of KS in the early clinical stages are likely to improve outcomes.

Clinical predictors of mortality on univariate logistic regression included visceral KS, fever, and sepsis. Advanced KS presents as multiple disseminated KS lesions with or without massive lymphedema and often accompanied with visceral involvement. When KS involves the viscera, commonly lungs and gastrointestinal tract, patients often have systemic life-threatening complications requiring admission and intensive care. Hence, the prognosis for advanced KS is usually poor.^[13]^ Fever is a common sign of sepsis; thus, most of our patients who were diagnosed with sepsis also had fever.

Medical interventions, including blood transfusion, oxygen, and intravenous antibiotics, were also associated with death on univariate logistic regression. As most of the patients who died were very ill, with advanced KS disease on admission, they were more likely to be commenced on these medical interventions. Therefore, these treatments were only predictors of mortality in the sense that they were likely to be initiated in very ill KS patients who had advanced disease.

Several laboratory parameters, including low hemoglobin, low platelets, high white cell counts, and high HIV-1 viral loads, were also significantly associated with mortality. Anemia is a risk factor for HIV infection, and has previously been associated with shorter survival in HIV-infected patients.^[14]^ The severe anemia in the individuals who died may be a result of the more advanced HIV disease and advanced KS. Thrombocytopenia has previously been reported in KS patients and supposedly results from platelet sequestration in the abnormal KS tumor vessels.^[15,16]^ Therefore, advanced disease with numerous KS lesions possibly explains why individuals who died had lower platelet counts. At the time of admission, patients who eventually died also had higher white cell counts above the upper limit of normal. Our findings are consistent with previous studies that have reported high white cell and neutrophil counts to be associated with a poor prognosis in the form of death or KS disease progression.^[17,18]^ Higher white cell counts may reflect a higher degree of chronic inflammation or may be a sign of sepsis in individuals who died. HIV viral load was 500-fold higher at baseline in individuals who eventually died than individuals who were discharged. HIV viral load is a marker of HIV disease control.^[19]^ In addition, poor control of HIV infection predisposes to KS development and progression.^[20]^ In our study cohort, individuals who died had a history of poor compliance to ART compared to those who were discharged from hospital. This may have contributed to the advanced disease presentation, high HIV viral load, and poor outcome. However, CD4 counts were not predictive of mortality.

After controlling for other predictors of mortality, only sepsis (or signs and symptoms of sepsis) was independently associated with mortality. Sepsis is common in immunosuppressed HIV patients. It has been previously identified as a major cause of admission and mortality in HIV patients.^[21]^ Unfortunately, blood cultures were not done to confirm the diagnosis of sepsis in our patients due to unavailability of resources. It is possible that the signs and symptoms of sepsis were due to a phenomenon called Kaposi sarcoma inflammatory cytokine response (KICS). KICS is common in severely ill KS patients, mimics severe sepsis, is associated with high Interleukin 6 (IL-6) production, associated with high KSHV viral loads, and is also associated with a high mortality rate.^[22–24]^ It is more probable that our patients had KICS and not sepsis. This is because empirical intravenous broad-spectrum antibiotics that are known to be effective in septic HIV individuals in our setting were administered in our patients with signs of sepsis; however, these antibiotics had no effect and even seemed to be associated with poor outcomes. Furthermore, there was no interaction between treatment with intravenous antibiotics and a diagnosis of sepsis. Prospective cohort studies and more extensive laboratory testing need to be conducted to address this issue.

## 5 Study limitations

We could not definitely differentiate sepsis vs KICS because blood cultures, KSHV viral loads, and IL-6 levels were not assessed during admission. A prospective cohort study would be required to definitively address this. In addition, the sample size was not powered enough to determine statistical differences in marginally significant variables including hemoglobin levels and creatinine on multivariate regression.

## 6 Conclusion

Advanced disease with visceral involvement, severe anemia, and symptoms and signs of sepsis were among the most common reasons for admission of epidemic KS patients. Several factors, including advanced KS clinical stage, sepsis, low hemoglobin, low platelet count, high white cell count, and high HIV viral load, were associated with mortality. However, only signs and symptoms of sepsis were independent predictors of mortality for admitted KS patients.

## Author contributions

**Conceptualization:** Owen Ngalamika.

**Data curation:** Faheema Vally, Wencilaus Margret Pious Selvaraj.

**Formal analysis:** Owen Ngalamika.

**Funding acquisition:** Owen Ngalamika.

**Investigation:** Faheema Vally.

**Methodology:** Faheema Vally, Owen Ngalamika.

**Resources:** Faheema Vally, Owen Ngalamika.

**Supervision:** Owen Ngalamika.

**Validation:** Wencilaus Margret Pious Selvaraj, Owen Ngalamika.

**Writing – original draft:** Faheema Vally.

**Writing – review & editing:** Faheema Vally, Wencilaus Margret Pious Selvaraj, Owen Ngalamika.
